# Neutrophil‐to‐lymphocyte ratio predicts hemorrhagic transformation in ischemic stroke: A meta‐analysis

**DOI:** 10.1002/brb3.1382

**Published:** 2019-08-20

**Authors:** Ruirui Zhang, Xiaodong Wu, Wenjie Hu, Li Zhao, Shoucai Zhao, Ji Zhang, Zhaohu Chu, Yang Xu

**Affiliations:** ^1^ Department of Neurology Wannan Medical College First Affiliated Hospital Yijishan Hospital Wuhu China; ^2^ Key Laboratory of Non‐coding RNA Transformation Research of Anhui Higher Education Institutes Wannan Medical College Wuhu China; ^3^ Non‐coding RNA Research Center of Wannan Medical College Wuhu China

**Keywords:** acute ischemic stroke, hemorrhage transformation, meta‐analysis, mortality, neutrophil‐to‐lymphocyte ratio

## Abstract

**Introduction:**

The neutrophil‐to‐lymphocyte ratio (NLR) has been shown to be a marker associated with inflammation and is independently associated with the adverse clinical outcomes of symptomatic intracranial hemorrhage, cancer, and cardiovascular disease. Hemorrhagic transformation (HT) is a serious complication of ischemic cerebral infarction and can be intensified by therapeutic interventions for acute ischemic stroke (AIS). The purpose of our research was to explore the predictive effect of NLR for HT in patients with AIS and to determine the best predictive value.

**Methods:**

PubMed, Web of Science, EMBASE, MEDLINE, Cochrane, and Google Scholar were searched. The primary endpoint was HT, and subgroup analysis was performed. Review Manager software version 5.3 was used to statistically analyze the outcomes.

**Results:**

A total of seven studies including 3,726 patients met the inclusion criteria. The pooled odds ratio (OR) value of the high NLR that predicted HT in AIS patients was 1.53 (95% CI, 1.21–1.92; *p* = .0003). In addition, 1.10 (95% CI, 1.05–1.15; *p* < .0001) was the pooled OR of the high NLR associated with increased 3‐month mortality in patients with AIS. In the subgroup analysis with an NLR cutoff value of 7.5–11, the correlation between NLR above the cutoff value and the rate of HT in patients with AIS was statistically significant (OR, 7.93; 95% CI, 2.25–27.95; *p* = .001).

**Conclusion:**

A high NLR can predict HT and 3‐month mortality in patients with AIS. Regardless of the country of origin and the sampling time, an NLR with a cutoff value of 7.5–11 was independently associated with HT in AIS patients.

## 1 INTRODUCTION

Acute ischemic stroke (AIS) is the leading cause of disability and is the main cause of death worldwide after cardiovascular diseases and cancer (Goldstein et al., 2011). Acute ischemic stroke is characterized by a sudden loss of neurological function in the affected area (Inanc & Inanc, 2018). However, currently, the best treatment for AIS is only reperfusion therapy, which includes intravenous tissue plasminogen activator (rt‐PA) and endovascular therapy (EVT; Brooks et al., 2014). Hemorrhagic transformation (HT) is the most serious complication for patients with AIS and can be induced by the natural evolution of AIS or by reperfusion therapy (Jickling et al., 2014; Nagaraja et al., 2018). Hemorrhagic transformation is classified as hemorrhagic infarction (HI) and parenchymatous hematoma (PH; von Kummer et al., 2015). Hemorrhagic transformation is thought to be a hemorrhage that occurs in the ischemic region, and on a computed tomography (CT) scan, it appears as a high‐density shadow in the low‐density region (Ge, Chen, Pan, Chen, & Zhou, 2018). Acute ischemic stroke strongly activates the immune system, and neutrophil counts have been demonstrated to be a main source of matrix metalloproteinase‐9 (MMP‐9) in patients with AIS and to contribute to the early destruction of the blood–brain barrier (Jickling et al., 2014). Lymphocyte count is a common health indicator that may be affected by acute physiological stress (Li, Cui, Ma, Ma, & Li, 2015). A relative decrease in lymphocytes is indicative of the cortisol‐induced stress response and the sympathetic tone (Acanfora et al., 2001), which leads to an increase in pro‐inflammatory cytokines, thereby aggravating ischemic injury (Park et al., 2011).

We aim to find an indicator of inflammation that predicts HT in order to provide a reference for the diagnosis and treatment of patients with AIS. Some research studies have reported that antineutrophil therapy has no clear clinical effect (Krams et al., 2003), but others have demonstrated that certain chemicals produced by the inflammatory response may contribute to an improvement in brain function after AIS (Spite & Serhan, 2010). Therefore, it should be understood that immunomodulatory therapy should be a balance between anti‐inflammatory and pro‐inflammatory responses in order to provide clinical value for the prognosis of patients with AIS (Xue et al., 2017). The neutrophil‐to‐lymphocyte ratio (NLR) represents a level of immunity as illustrated by the balance between neutrophils and lymphocytes (Guo et al., 2016). Moreover, the NLR has been shown to be an indicator of the inflammatory state of individuals and has been reported to predict the accuracy of clinical outcomes in patients with AIS (Lattanzi et al., 2018).

However, the association between the NLR and HT in AIS is still under discussion. Previous studies have shown that the NLR is independently associated with clinical outcomes and short‐term mortality in patients with AIS (Gokhan et al., 2013; Tokgoz, Keskin, Kayrak, Seyithanoglu, & Ogmegul, 2014; Yu et al., 2018; Zhang et al., 2017). In addition, it was reported that a higher admission NLR is an independent risk factor for HT and 3‐month functional outcome in patients with AIS both with and without reperfusion therapy (Duan et al., 2018; Goyal et al., 2018; Guo et al., 2016; Maestrini et al., 2015; Pikija et al., 2018; Song et al., 2018). The subject of our study was to comprehensively summarize the value of NLR for predicting HT in patients with AIS by performing a meta‐analysis.

## 2 MATERIALS AND METHODS

### 2.1 Search strategy

Relevant articles published from 1 January 2010 to 30 May 2019 were searched in major databases such as PubMed, Web of Science, EMBASE, MEDLINE, Cochrane, and Google Scholar. We used the following keywords for literature searches: “NLR”, “neutrophil lymphocyte ratio”, “Acute Ischemic Stroke”, “AIS”, “Ischemic Stroke”, “Stroke”, “Hemorrhage Transformation”, and “HT”.

### 2.2 Inclusion and exclusion criteria

Articles were included if the following inclusion criteria were met: (a) The study subjects were patients diagnosed with AIS; (b) white blood cell counts and NLRs were calculated after admission and before reperfusion therapy; (c) the best predictive value of NLR was provided to predict HT as the endpoint event; and (d) the odds ratio (OR) and 95% confidence interval (CI) for HT or mortality could be calculated from the data provided in the paper. The following are the relevant exclusion criteria: (a) Experimental subjects were animals; (b) patients had diseases not associated with AIS; (c) the endpoint event of the study was not HT; and (d) NLR was not a risk factor for HT in AIS patients.

### 2.3 Data extraction and risk of bias in the included studies

Two independent investigators (Ruirui Zhang and Wenije Hu) selected the studies that met the above inclusion criteria. One investigator (Yang Xu) was responsible for solving vague or divergent problems. We extracted relevant research data during the search process, including the first author's name, year of publication, country, number of patients, patient source, median or mean age of patients, research method, patient characteristics, the best predicted value of NLR, blood sampling time, and OR and 95% CI for HT and 3‐month mortality. There are different classification and classification criteria for HT in AIS patients, such as HT1, HT2, PH1, and PH2. All studies included in our meta‐analysis defined the symptomatic intracranial hemorrhage (sICH) as the study subject. If univariate and multivariate regression analyses were available in the study, multivariate regression analysis data were used for OR calculations. The bias risk for each analysis was independently assessed by the risk of bias graph and the risk of bias summary generated by the Cochrane Collaboration.

### 2.4 Statistical analysis

Forest and funnel plots were used to analyze the prognostic role of NLR for HT in AIS patients, and the Review Manager version 5.3 software from Cochrane was used to test the publication bias generated in the study. The log(OR) and its standard error were calculated by OR and 95% CI and used for merging. The OR and 95% CI were pooled to analyze the association of the NLR with HT and 3‐month mortality. This study also assessed the heterogeneity between the studies. If *I* ^2^ > 50% or *p* < .10, meaning significant heterogeneity, a random‐effects model was used; otherwise, a fixed‐effects model was adopted. The subgroup analysis was used to find the cause of heterogeneity and minimize it. A *p* value <.05 was considered statistically significant.

## 3 RESULTS

### 3.1 Study inclusion

**Figure 1:**
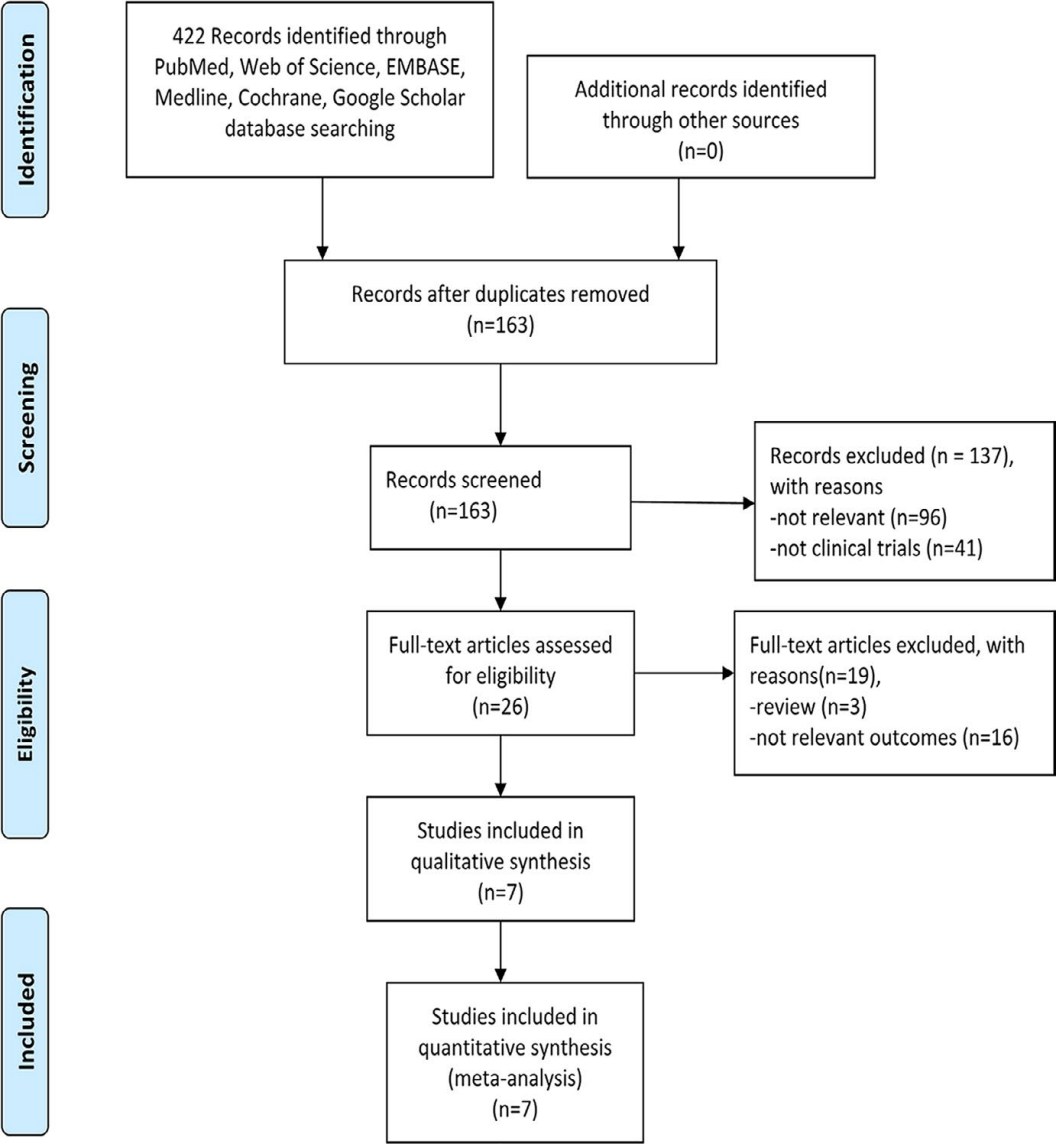
Flow diagram of study retrieval and screening

The flowchart of the research design is shown in Figure 1. After the initial keyword literature search, 422 articles were included. A total of 163 studies were retrieved after deleting duplications. Then, we excluded 137 papers because they were not relevant (*n* = 96) and were not clinical trials (*n* = 41). Of the remaining 26 articles, 19 were removed due to being reviews (*n* = 3) and not reporting relevant outcomes (*n* = 16). Seven articles were eventually included in our meta‐analysis (Duan et al., 2018; Goyal et al., 2018; Guo et al., 2016; Maestrini et al., 2015; Malhotra et al., 2018; Pikija et al., 2018; Song et al., 2018).

### 3.2 Study characteristics

All seven studies included were published between 2015 and 2019. Three of them were from China, two from the United States, one from France and Finland, and one from Austria. The best cutoff NLR value for HT from each article was different, ranging from 3.89 to 10.59. The OR and 95% CI of NLR as the predicted value for HT were obtained from multivariate regression analyses, and all ORs were adjusted. The basic characteristic features of the patients enrolled in the seven studies are summarized in Table 1.

**Table 1:** Basic statistical characteristics of included studies

Author	Year	Country	Research method	*N* (F/M)	Patient characteristics	Age, year	Best predicted value of NLR	Time of blood collection	Type	HT (OR)	Mortality (OR)	Adjusted OR
Ilaria Maestrini	2015	France and Finland	Prospective	846 (416/430)	AIS patients after rt‐PA	Median 71	≥4.8	On admission	sICH	3.71 (1.97–6.98)^a^	1.08 (0.91–1.28)^a^	Yes
Zhiliang Guo	2016	China	Prospective	189 (66/123)	AIS patients after rt‐PA	Mean 65.0	≥10.59	Admission, 3–6 hr after rt‐PA, 12–18 hr after rt‐PA, and 36–48 hr after rt‐PA	sICH	7.93 (2.25–27.99)^a^	NR	Yes
									PH	8.50 (2.69–26.89)^a^		
Zhenhui Duan	2018	China	Retrospective	616 (248/368)	AIS patients after EVT	Median 66	≥7	Before EVT	sICH	1.84 (1.09–3.11)^a^	1.57 (0.94–2.65)^a^	Yes
Slaven Pikija	2018	Austria	Retrospective	187 (101/86)	AIS patients after EVT	Median 74	≥3.89	On admission	sICH	1.09 (1.00–1.20)^a^	NR	Yes
Nitin Goyal	2018	America	Retrospective	293 (146/147)	AIS patients after MT	Mean 62	≥6.62	≤24 hr	sICH	1.11 (1.03–1.20)^a^	1.08 (1.01–1.16)^a^	Yes
Quhong Song	2018	China	Retrospective	938 (337/601)	AIS patients	Mean 64.10	≥4.5	≤24 hr	sICH or PH	1.97 (1.33–2.92)^a^	NR	Yes
Konark Malhotra	2018	USA	Retrospective	657 (328/329)	AIS patients after rt‐PA	Mean 64.3 ± 14.4	＜2.2	≤12 hr	sICH	NR	1.12 (1.04–1.20)	No

Abbreviations: HT, hemorrhage transformation; *N* (F/M), number of patients (female/male); NLR, neutrophil‐to‐lymphocyte ratio; NR, not reported; OR, odds ratio; PH, parenchymal hematoma; sICH, symptomatic intracranial hemorrhage; y, year.

a Multivariate regression analysis.

### 3.3 Overall efficacy

**Figure 2:**
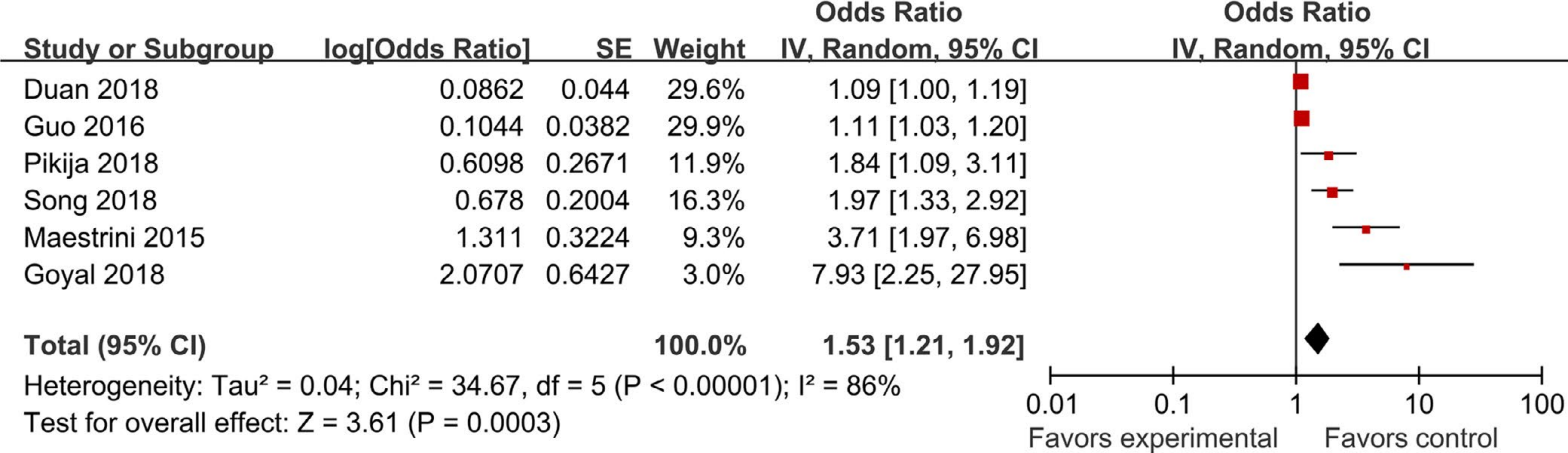
Pooled odds ratio of NLR above the cutoff value for HT in patients with AIS

The seven studies including 3,726 patients indicated that NLR above the optimal cutoff value as a risk factor for HT in AIS. We pooled the data and found that using the random‐effects model, NLR above the optimal cutoff value was associated with HT rates in patients with AIS, with a pooled OR of 1.53 (95% CI, 1.21–1.92; *p* = .0003, Figure 2). The heterogeneity detected between the seven articles was *I* ^2^ = 86%, *p* < .00001. Acute ischemic stroke patients with higher NLRs were 1.53 times more likely to develop HT than those with lower NLRs. A significant correlation between NLR and 3‐month mortality in AIS patients is shown in Figure S1 available online, with a pooled OR of 1.10 (95% CI, 1.05–1.15; *p* < .0001). The heterogeneity detected between the four papers was *I* ^2^ = 0%, *p* = .49.

### 3.4 Subgroup analyses

**Figure 3:**
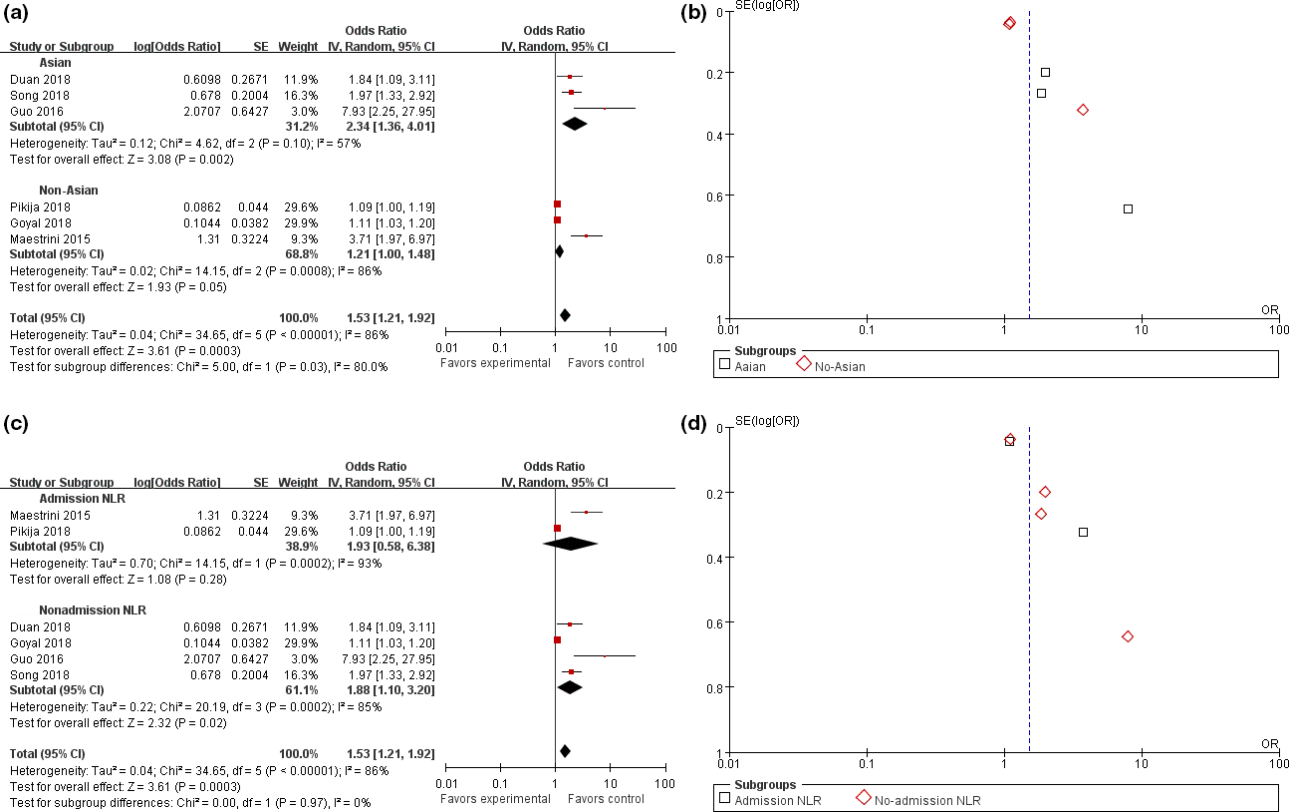
Forest and funnel plot of Asian and non‐Asian populations and the time of laboratory examination subgroup analysis

**Figure 4:**
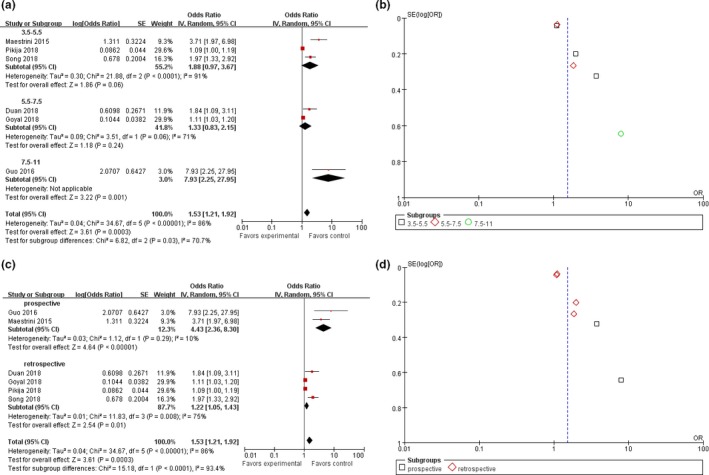
Forest and funnel plot of the best cutoff value and the research method subgroup analysis

**Figure 5:**
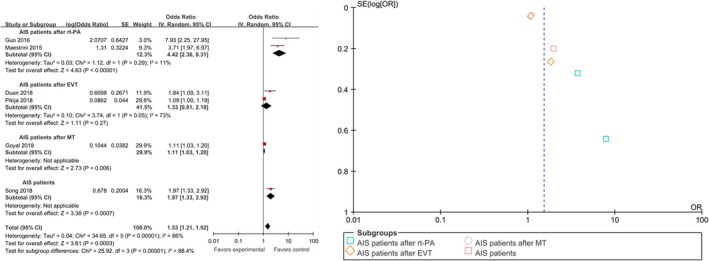
Forest and funnel plot of characteristics of the research object subgroup analysis

We performed a subgroup analysis of six studies using HT as the outcome measure. A country‐based subgroup analysis showed that the effect of high NLR on HT rates in Asia was statistically significant, with an OR of 2.34 (95% CI, 1.36–4.01; *p* = .002) and an OR of 1.21 (95% CI, 1.00–1.48; *p* = .05) in non‐Asian countries (Figure 3a,b). Subgroup analysis based on time of laboratory examination as presented in Figure 3c,d showed that the pooled OR for HT was 1.93 (95% CI, 0.58–6.38; *p* = .28) in the admission group and 1.88 (95% CI, 1.10–3.20; *p* = .02) in the nonadmission group. Of all the articles, three reported an NLR cutoff value of 3.5–5.5, and the pooled OR for the HT rate was 1.88 (95% CI, 0.97–3.67; *p* = .06). The NLR cutoff value reported in the other two articles ranged from 5.5 to 7.5, and the pooled OR for the HT rate was 1.33 (95% CI, 0.83–2.15; *p* = .24). The last article reported an NLR cutoff value of 7.5 with an OR for HT of 7.93 (95% CI, 2.25–27.95; *p* = .001; Figure 4a,b). In addition, the pooled OR was 4.32 (95% CI, 2.46–7.60; *p* < .00001) for the two prospective studies and 1.22 (95% CI, 1.05–1.43; *p* = .01) for the four retrospective studies (Figure 4c,d). The subjects of two articles were AIS patients after thrombolysis, and the pooled OR was 4.42 (95% CI, 2.36–8.31; *p* < .00001); the subjects of another two studies were AIS patients after EVT, and the pooled OR was 1.33 (95% CI, 0.81–2.18; *p* = .27); the subjects of one other paper were AIS patients after MT, and the pooled OR was 1.11 (95% CI, 1.03–1.20; *p* = .006); and the subjects of the last article were AIS patients, and the pooled OR was 1.97 (95% CI, 1.33–2.92; *p* = .0007; Figure 5). All the pooled results above are described in Table S1 available online.

### 3.5 Risk of bias in the included studies

We used the Cochrane bias risk tool to evaluate the quality of the seven included studies. The risk of bias graph and the risk of bias summary are shown in Figures S2 and S3 available online, which show that the risk of bias in the included articles is low. This meta‐analysis indicates that the studies show publication bias (online Figure S4). Except for Malhotra et al.'s study, we did not perform a regression analysis on HT.

## 4 DISCUSSION

This research study was designed to assess the predictive value of NLR for HT in patients with AIS and to determine the best predictor of NLR. The meta‐analysis of the seven studies that met the inclusion criteria showed that a higher NLR was independently associated with HT in patients with AIS, regardless of reperfusion treatment. Based on these results, understanding the mechanisms of NLR and HT may help identify high‐risk HT populations, thereby providing safer and better clinical use of therapeutic interventions.

Inflammation is considered a major player in the development of secondary brain injury after stroke (Qun et al., 2017; Ye et al., 2017). The neutrophil‐to‐lymphocyte ratio has been recognized as a simple and reliable inflammatory marker (Zahorec, 2001). The destruction of the blood–brain barrier has been identified as a potential mechanism for HT after ischemic stroke, and neutrophils have been shown to be an important source of MMP‐9, which can lead to early disruption of the blood–brain barrier in AIS (Hao et al., 2017). Previous studies have revealed a strong neutrophil infiltration in the infarcted and hemorrhagic areas (Rosell et al., 2008). In addition, pro‐inflammatory N1 neutrophils contribute to cerebral edema and have neurotoxic effects, while anti‐inflammatory N2 neutrophils have been shown to reduce this excessive immune response, thereby allowing neurons to survive (Hermann, Kleinschnitz, & Gunzer, 2018).

Lymphocytes accumulate in the ischemic brain 3–6 days after stroke, which is later than neutrophils (Li et al., 2005). Neutrophils and lymphocytes have different predictive value for the prognosis of acute cerebral infarction, although higher neutrophils can cause heavier initial stroke, but lower lymphocytes are associated with poorer long‐term prognosis (Kim et al., 2012). One of the mechanisms may be that the reduction in lymphocyte count represents an acute stress response in humans, and another may be that the relative reduction in lymphocytes is a marker of increased prestroke cortisol levels and sympathetic tone, which cause an increase in pro‐inflammatory factors, thereby aggravating ischemic injury (Acanfora et al., 2001; Ommen, Gibbons, Hodge, & Thomson, 1997; Park et al., 2011). Therefore, high NLR in peripheral blood predicts poor prognosis in patients with AIS (Fang et al., 2017; Hermann et al., 2018).

Although the articles we included indicated that high NLR is a predictor of HT in patients with AIS, the specific predictive value of NLR varies from study to study. Our target is determining the best NLR predictors. The cutoff values for various diseases vary; for example, the symptomatic intracranial hemorrhage (sICH) has a value of 4.08–7.35 (Ye et al., 2017), and cardiovascular disease has a value of 3.5–7.6 (Bhat et al., 2013). In one of the articles we included, Malhotra et al. (2018) did not perform a regression analysis on the predictive value of NLR in patients with AIS, and we excluded it. In the remaining analyses, the best cutoff value for NLR for predicting HT in AIS patients ranged from 3.89 to 10.59. Three of the articles reported the best cutoff value to be approximately 4.5, and the other three studies reported a cutoff value of more than 6. Predicting the NLR cutoff value for HT may be affected by many other external factors, such as blood sample time, different biochemical analyzers, individual differences, and country of origin.

Our study found that a higher NLR cutoff value has a better predictive power for HT in AIS patients. Maestrini et al. (2015) showed that a higher NLR was independently associated with sICH in AIS patients after rt‐PA with a cutoff value of 4.8. Goyal et al. (2018) reported that higher NLR is an independent predictor for sICH in AIS patients after mechanical thrombectomy (MT) with a cutoff value of 6.62. Duan et al. (2018) described that a higher NLR (predictive value NLR ≥ 7) was independently associated with the onset of sICH after EVT. Furthermore, Guo et al. (2016) proved that NLR is a dynamic variable, and its variation is associated with HT after thrombolysis in AIS with a high cutoff value of 10.59, which had a high area under the curve (AUC) of 0.814 and a high sensitivity of 76.5%. Song and colleagues also demonstrated that a higher NLR with a cutoff value of 4.5 (sensitivity 64.8% and specificity 60.1%) is associated with a greater risk of HT in patients with AIS (Song et al., 2018). A subgroup analysis in our study showed that the risk of HT in AIS patients with an NLR cutoff value of 7.5–11 was 7.93 times higher than that of the control group. If the NLR of the patient was <7.5, then the predictive value of HT was not as good. Malhotra et al. focused on the absence of significant differences between NLR < 2.2 and HT in patients with AIS, and the study was not included in our subgroup analysis. Compared with our results, it may be that the NLR value reported in that study was too small, so it was not as predictive, and on the other hand, this difference verified the reliability of our results. We found that NLR with a cutoff value of 7.5–11 was able to predict HT in patients with AIS, regardless of other factors, such as the sampling time of NLR in the emergency department.

The literature included in this study was independently associated with HT in AIS patients after adjusting for confounding factors. However, there was significant heterogeneity in the six studies. Subgroup analysis was used to reduce or find sources of heterogeneity. In the admission group, a higher NLR was found to be associated with HT, but the association was not significant among the nonadmission group. However, this conclusion should be treated with caution, since the number of studies in each subgroup was limited, especially in the admission group. We also divided the studies according to the study methods. The pooled OR of the two prospective studies was higher than that of the four retrospective studies (4.32 vs. 1.22, respectively).

There are several limitations in our meta‐analysis. First, the sample size included in the study is small, including only seven articles, which can have an impact on the results. The subgroup analysis had even fewer studies, so caution should be given to the subgroup results. Moreover, a random‐effects model determined that the heterogeneity between the studies was significant, which could be indicative of publication bias. Finally, the NLR cutoff value between the studies varied. Therefore, more studies are needed to validate the prognostic value of NLR for HT in AIS patients. Based on the time of blood sampling and patient characteristics, larger sample size and higher quality sample studies are needed in the future to determine the association between NLR and HT in AIS patients. In addition, further investigation is needed to dynamically monitor the NLR and explore changes in NLR over time to predict HT in AIS patients.

## 5 CONCLUSION

In conclusion, NLR is a promising inflammatory indicator that can effectively predict the clinical outcome of HT in patients with AIS. The results of this meta‐analysis showed that NLR with a cutoff value of 7.5–11 was a predictor of HT rate and 3‐month mortality in patients with AIS, regardless of country and sampling time.

## CONFLICT OF INTEREST

The authors have no conflicts of interest to declare.

## Data Availability

The data that support the findings of this study are available from the corresponding author upon reasonable request.
