# Supplementary figures and images for: Neutrophil‐to‐lymphocyte ratio predicts hemorrhagic transformation in ischemic stroke: A meta‐analysis
Source: Brain Behav. 2019 Aug 20;9(9):e01382. doi: 10.1002/brb3.1382 (PMC6749596; doi:10.1002/brb3.1382)

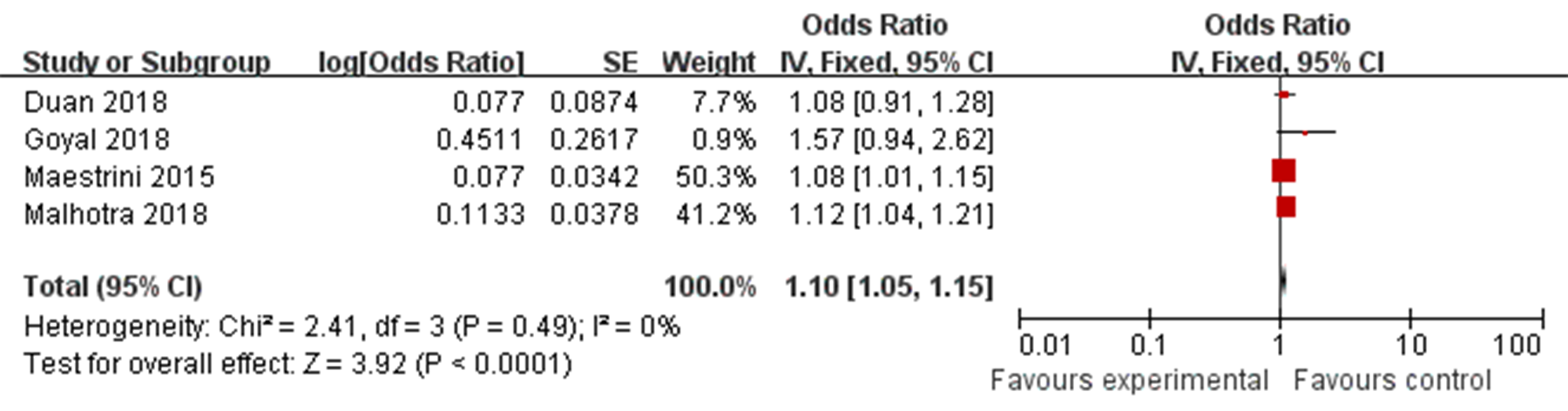

Supplement: Supplementary file 1 [file BRB3-9-e01382-s001.tif]

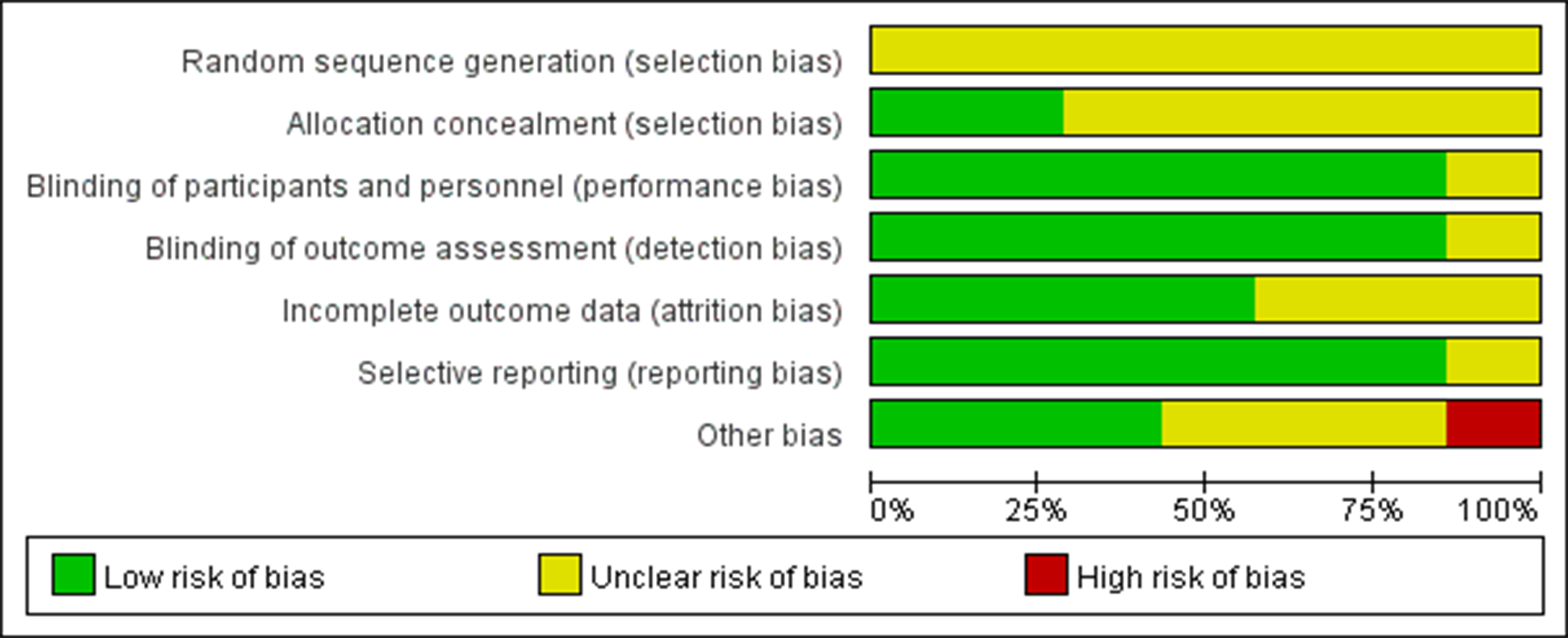

Supplement: Supplementary file 2 [file BRB3-9-e01382-s002.tif]

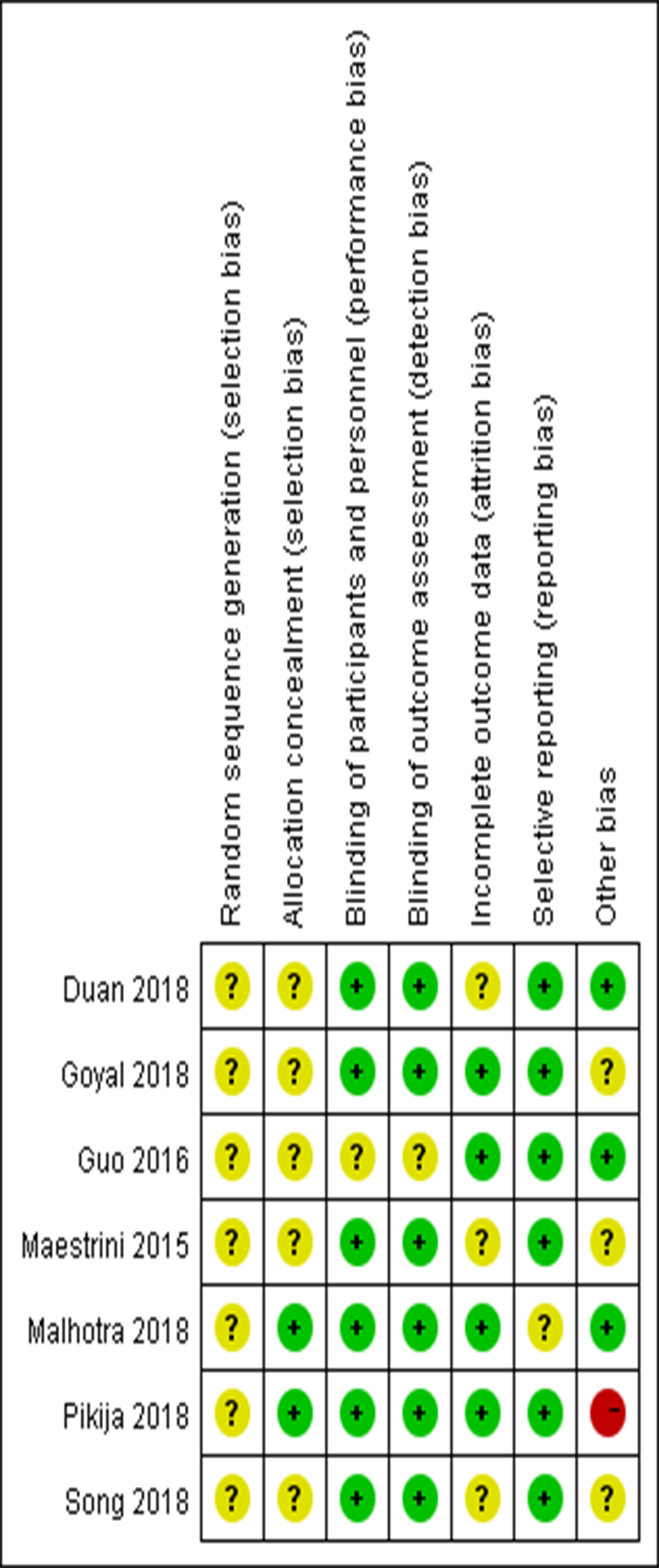

Supplement: Supplementary file 3 [file BRB3-9-e01382-s003.tif]

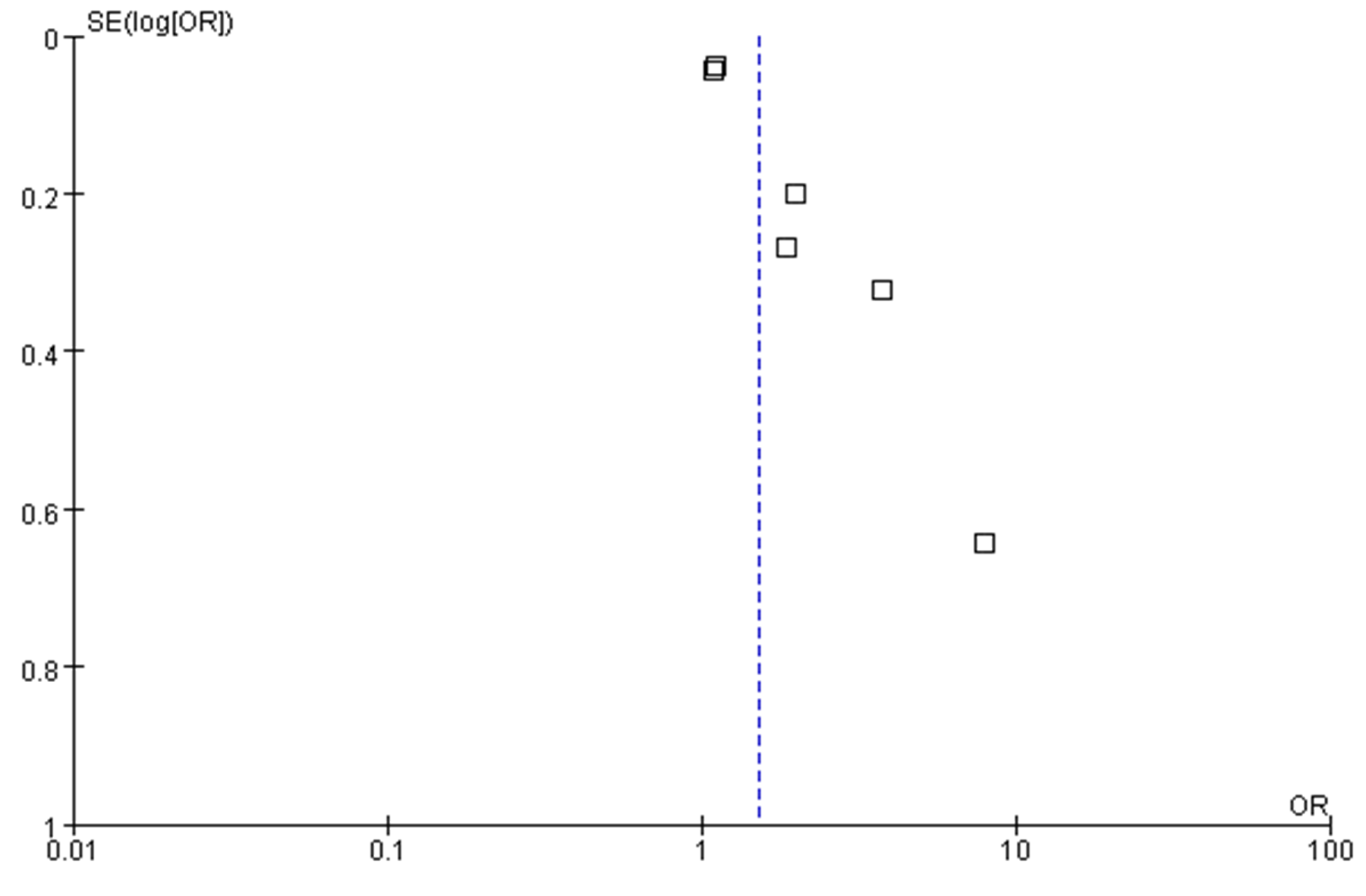

Supplement: Supplementary file 4 [file BRB3-9-e01382-s004.tif]
