# Supplementary material for: Neutrophil‐to‐lymphocyte ratio predicts hemorrhagic transformation in ischemic stroke: A meta‐analysis
Source: Brain Behav. 2019 Aug 20;9(9):e01382. doi: 10.1002/brb3.1382 (PMC6749596; doi:10.1002/brb3.1382)
Supplement: Supplementary file 5 [file BRB3-9-e01382-s005.docx]

Supplementary Table 1. Subgroup analysis results of HT rate.

| Groups | N | Model | Pooled OR (95%CI) | P | Heterogeneity (P, I2) | Conclusion |
| --- | --- | --- | --- | --- | --- | --- |
| Total | 6 | Random | 1.53 (1.21-1.92) | 0.0003 | p<0.00001,86% | Positive |
| Asian | 3 | Random | 2.34 (1.36, 4.01) | 0.002 | 0.10,57% | Positive |
| No Asian | 3 | Random | 1.21 (1.00, 1.48) | 0.05 | 0.0008,86% | Positive |
| Admission NLR | 2 | Random | 1.93 (0.58, 6.38) | 0.28 | 0.0002,93% | Negative |
| Non-admission NLR | 4 | Random | 1.88 (1.10, 3.20) | 0.02 | 0.0002,85% | Positive |
| 3.5＜NLR≤5.5 | 3 | Random | 1.88 (0.97, 3.67) | 0.06 | P < 0.0001,91% | Negative |
| 5.5＜NLR≤7.5 | 2 | Random | 1.33 (0.83, 2.15) | 0.24 | 0.06,71% | Negative |
| 7.5＜NLR≤.11 | 1 | - | 7.93 (2.25, 27.95) | 0.001 | - | Positive |
| prospective | 2 | Fixed | 4.32 (2.46, 7.60) | P < 0.00001 | 0.29,10% | Positive |
| retrospective | 4 | Random | 1.22 (1.05, 1.43) | 0.01 | 0.008,75% | Positive |
| AIS patients after rt-PA | 2 | Fixed | 4.42 (2.36, 8.31) | p< 0.00001 | 0.29,11% | Positive |
| AIS patients after EVT | 2 | Random | 1.33 (0.81, 2.18) | 0.27 | 0.05,73% | Negative |
| AIS patients after MT | 1 | - | 1.11 (1.03, 1.20) | 0.006 | - | Positive |
| AIS patients | 1 | - | 1.97 (1.33, 2.92) | 0.0007 | - | Positive |

N: number, CI: confidence interval, OR: odds ratio, NLR: Neutrophil-to-Lymphocyte
